# A Qualitative Content Analysis of Cardiovascular Diseases Related Health Information Targeted at the Hui Minority on Chinese WeChat Official Accounts

**DOI:** 10.3390/healthcare9101359

**Published:** 2021-10-13

**Authors:** Lei Yang, Yuping Mao, Jeroen Jansz

**Affiliations:** 1 Erasmus Research Centre for Media, Communication and Culture, Erasmus School of History, Culture and Communication, Erasmus University Rotterdam, 3062 PA Rotterdam, The Netherlands; jansz@eshcc.eur.nl; 2 Communication Studies, California State University Long Beach, Long Beach, CA 90840, USA; Yuping.Mao@csulb.edu

**Keywords:** cardiovascular diseases, health information, WeChat official accounts, Chinese Hui ethnic minority people, qualitative content analysis, traditional Chinese medicine

## Abstract

In this study, we focus on the information available in WeChat official accounts about cardiovascular diseases (CVDs), which are a leading cause of death in China. We are particularly interested in information targeting the Chinese Hui minority people, who have a high prevalence of cardiovascular risk factors (CVRFs). Our exploratory research therefore investigates whether and how the articles on WeChat official accounts are targeted at the Hui people. We used a qualitative approach to analyze 108 articles. Two related themes emerged: descriptions of how to live a healthy life; and explanations of CVDs and CVRFs. Traditional Chinese medicine likewise surfaced from the analysis as a specific and unique theme in the Chinese social and cultural context. Despite the high prevalence of CVRFs among the Hui, none of the articles included information tailored to them.

## 1. Introduction

Social media has become a prominent channel for health communication [1,2,3] because of its effectiveness in reaching a global public [2,3]. Different kinds of health information is disseminated on such platforms, and people can search for health information based on their own preferences, making it a way to communicate with people about their health issues [4,5].

The rise of social media in China offers the general public new opportunities to communicate online. WeChat in particular is very popular and is increasingly used to share and seek health information [6]. WeChat is an instant messaging application for smartphones and has several features such as video calls, official accounts, moments, games, and WeChat Pay services [7]. WeChat official accounts normally focus on particular topics and are run by different agents, including members of the public, media companies, businesses, and the government. Some of these accounts are aimed at benefitting financially those who run or appear on them, whereas others are not-for-profit services. WeChat users can choose to follow their preferred official accounts to get health information and advice. In terms of the dissemination of such information, the main difference between the platform’s messaging and official accounts is that the former is mainly used by individuals to communicate and interact with their own social networks, whereas the latter regularly posts articles and messages to followers. The official accounts are a new and better way to distribute health information using IT that supports multimedia messages [8]. In this study, we focus on information related to cardiovascular diseases (CVDs) posted in WeChat official accounts, as these are the leading cause of death in China [9,10,11].

Hypertension, diabetes, dyslipidemia, overweight/obesity, and smoking are known to be the five main cardiovascular risk factors (CVRFs) [9,10,11,12]. There are differences in how prevalent these are in various Chinese ethnic groups. The Hui, a Chinese Muslim minority, have a higher incidence of CVRFs than most other ethnic groups [9], which can be attributed to their culturally different lifestyles and eating habits [13]. Specifically, Wu et al. [9] reported that the Hui minority is more likely than Han residents to have ≥1, ≥2, or ≥3 CVRFs, and concluded that there is high prevalence and clustering of CVRFs among the Hui minority. Furthermore, our previous research revealed that the Hui participants in our survey and focus groups have a strong and unique need for health information related to preventing and treating CVDs [14,15,16], but face difficulties in obtaining it because of a lack of access or availability [15]. Accordingly, we now want to explore whether and how the articles on WeChat official accounts target this minority group.

According to social cognitive theory (SCT) [17,18,19], media content is vital because it has an impact on recipients. Highly circulated, relevant CVD content on WeChat official accounts could therefore have a major effect on the health beliefs and behaviors of Chinese users of the platform. Consequently, a content analysis of the health information available on these accounts could provide meaningful insights into how to design effective and culturally sensitive health education programs for the Hui minority. This study therefore briefly introduces the theoretical background and relevant literature, describes the content analysis method used, and presents the key findings. The results are then discussed within their theoretical and social contexts, and the study’s conclusion includes practical suggestions and future research directions.

## 2. Literature Review

### 2.1. Healthcare System in China

China has a growing need to address issues concerning rapidly growing populations with CVDs [20]. Although the country has made progress in improving its citizens’ access to health services, it still faces the challenge of providing high-quality healthcare [20].

The healthcare system in China is complicated. Contrary to practices in most Western countries, where patients can consult a general practitioner for minor health issues, China’s public hospitals take care of both outpatient consultations and hospital admissions [21]. There are large public state-owned hospitals, private hospitals, clinics, and foreign healthcare providers [22]. A small number of state-owned hospitals dominate the industry, meaning that the other providers face difficulties in entering the market [22]. Patients generally prefer to consult doctors in the state-owned hospitals, because they consider these to be reliable [22]. The government has, however, reduced funding for these hospitals, meaning that they do not have enough money to pay their doctors, who therefore rely on prescribing very profitable medications to increase their income [22]. The conflicts between doctors and patients, as well as the high cost of treating chronic diseases, including CVDs, and the low quality of the healthcare supply, are the main healthcare issues currently facing China [21,23]. The result of this is that the healthcare available is subject to a great deal of criticism [22].

Health insurance also plays a role in how patients with chronic diseases utilize healthcare. It is evident that an increased reimbursement cap for chronic disease coverage is associated with reduced hospitalization rate among patients with both diabetes and hypertension [24]. Therefore, increasing the reimbursement cap for individuals with chronic conditions could help them to reduce adverse health impacts. In the latest healthcare reform, the Chinese central government mandated an increase in health insurance benefits for patients with chronic diseases at the outpatient level, which aims at encouraging regular clinical visits, adherence to treatment, and reducing hospitalizations [25].

### 2.2. WeChat and Health Communication

WeChat is the most widely and frequently used social media platform in China and plays an important role in the daily lives of the Chinese people [6]. WeChat was developed by the Chinese Tencent Company in 2011. It was first marketed in China as Weixin, but was then rebranded as WeChat in 2012 for international users [26]. The figures on Statista (2019) show that it is one of the world’s leading social networks, ranking fifth in terms of the number of active users globally, just behind Facebook, YouTube, WhatsApp, and Facebook Messenger [27]. As of August 2019, WeChat had over 1.13 billion active monthly users [26].

As a social media platform, WeChat covers a variety of topics, including health, and is regarded as an important health communication tool [8,28]. Nearly all WeChat users (98.35%) are exposed to such information, and about a third of them actively and frequently consume it [6]. Previous research has shown that the three most commonly used channels for information acquisition are WeChat moments, WeChat official account notifications, and group chats on WeChat [6]. WeChat moments is similar to a personal Facebook page where the user can post texts and share pictures and articles. The user’s friends can like or comment on these posts. WeChat official accounts are mostly from celebrities, famous brands, governments, businesses, universities, etc. A WeChat official account resembles a public Facebook page where users can follow the account and get notifications when this account publishes information. WeChat subscription accounts and WeChat service accounts are two typical types of WeChat official accounts [29]. WeChat official accounts are reported to be the second principal way of receiving health information after WeChat moments [6]. We therefore focused on these official accounts, in particular the service and subscription accounts that make up the majority.

Theories and empirical studies on media effects underline the importance of examining health-related content on media platforms. According to SCT, knowledge acquisition is affected by different forms of media [17]. In 2016, Valkenburg et al. published an overview of media effects research that also included the impact of mediated health campaigns [18]. Their analysis confirmed the influence that such campaigns have on health behaviors [18,30]. Social media has also been proven to be an effective tool for health interventions for many diseases, including cardiovascular conditions [31]. The innovative and various features of WeChat have turned it into a platform with great potential to affect individual and public health [6]. As an example, previous research revealed that WeChat official accounts have been used successfully to deliver malaria education interventions [8]. Given such health effects, our first research question centers on CVD-related health content in articles published by WeChat official accounts:

RQ1. What kinds of CVD-related health information are covered in the highly circulated articles on WeChat official accounts?

Our analysis is only concerned with the information communicated to users via articles from such accounts. We chose these articles from official accounts with high circulation, as they are more likely to have an impact on a wide range of users. We did not, however, study the effects of the health information on consumers, that is, WeChat users. Consequently, although some articles include comments from users, we did not incorporate them in our analysis.

### 2.3. CVD-Related Health Information Targeted at the Hui Minority Group in China

CVDs are the most common cause of death globally, placing a huge economic burden on society [9,11,32]. Relevant health education is therefore essential, and countries have used different health communication programs to help prevent these conditions. Prevention largely relies on the effective reduction of CVRFs, particularly by controlling the use of tobacco [13,32], encouraging physical activity (e.g., walking) [13,33], and promoting healthy diets [13,34]. Although many prevention principles apply to the general population, scholars using a cultural sensitivity approach recommend that interventions also take differences between ethnic groups into account. This perspective emphasizes the importance of considering the cultural factors affecting specific groups when creating appropriate health messages [35,36,37]. The American Heart Association, for example, proposed the development of special CVD-related interventions for minority populations [13], as these groups may have their own unique dietary habits and lifestyles that cause different health outcomes than the majority.

China is multicultural and multiethnic [38], but is dominated by the Han culture, which is reflected in the country’s social and healthcare systems. Previous research has shown that health communication in a multicultural society mainly takes the dominant culture into account, often neglecting those of non-dominant groups [35,39,40,41]. As a consequence, there are disparities in the health information provided to majority and minority populations [35,39,41,42,43].

Our second research question aims to examine how WeChat official accounts create CVD-related health information targeting the Hui ethnic minority group. The Hui are the third largest minority population in China and live all over the country [44]. They have their own ethnic culture, with many of them adopting Islamic dietary laws, which are different to the diets of the Han majority [44]. However, the Hui experience difficulties in obtaining health information they need [15].

Although social media cannot eliminate all the difficulties in accessing health information, they can provide better access to health programs or services for those in need (e.g., minority groups) than other mediated sources [2]. There is a high prevalence of CVRFs in the Hui population in China, making it important for this group to have access to relevant health information. Thus, it is important to investigate whether there is health information on WeChat official accounts targeted at the Hui and how the information targets them. The findings will be helpful for health promoters to diffuse health information among the Hui.

We therefore wanted to know whether and how WeChat official accounts target the Hui when presenting CVD-related health information. Our second research question is:

RQ2. Is CVD-related health information targeted at the Hui people covered in highly circulated articles on WeChat official accounts, and if so, how?

## 3. Methods

Our research questions focus on the health content available on WeChat official accounts, specifically the written text in their articles. Previous research has shown that qualitative content analysis is an effective method for analyzing the health-related content presented online, including via social media [45,46]. Our study follows the example of previous research on, for instance, information about weight loss surgery that is presented in online news media [45], and tweets from health professionals on Twitter [46].

### 3.1. Sample

This research is part of a larger project that used a survey and focus groups in Shenyang City from December 2016 to February 2017. In our survey study, we examined how the Hui people accessed and evaluated health information related to CVDs from different sources. In our focus groups study, we investigated which health needs the Hui people had related to CVDs. To be correspondent with our previous two studies, we chose to focus on the same time period (December 2016 to February 2017) in this study.

The qualitative content analysis in the current study included articles on WeChat official accounts. To collect our data, we used the Qingbo Index from Qingbo Big Data, which is the largest third-party evaluation platform for new media in China [47,48]. The company was founded in October 2014 and was the initiator of the Public Welfare World Internet Conference Network. To identify relevant articles, in February 2018 we entered the terms “heart attack”, “CVDs”, “diabetes”, “obesity”, “hypertension”, and “hyperlipidemia” in Chinese in the search engine of the Qingbo Big Data website (http://www.gsdata.cn/, accessed on 9 May 2018). We selected articles with a reading rate of over 50,000 (5W+), which means the article has been read over 50,000 (5W+) times, because this is considered to be a comparatively high figure for WeChat articles [49]. We eventually downloaded 158 articles from Qingbo, but 50 were either irrelevant (e.g., some were about pet health) or duplicates when we included different keywords in our search. Ultimately, 108 articles were downloaded in PDF format and analyzed.

### 3.2. Analysis

The unit of analysis was an individual article. A thematic analysis was conducted to analyze the CVD-related health content, as this is a systematic procedure with enough flexibility to allow for (theoretical) freedom in the interpretation [50]. We took an etic approach in our content analysis. The themes we drew from the articles were primarily based on salience and repetition. There could be both misinformation and scientific facts on CVDs and CVRFs that are heavily promoted in WeChat content. This dominant, prevalent, popular information is likely to reach more users. Our research focused on which health information/misinformation is promoted via WeChat official accounts and how.

To ensure the quality of the coding, one third of the articles were coded by two independent coders. After both researchers coded this selection, the codes of the two coders were compared and discussed. The result shows that the codes had 80% similarity, indicating a high level of agreement between the coders in interpreting the data. After the initial codes were established, all the remaining articles were hand-coded by the lead researcher.

We approached the data analysis thematically. The coding process was stepwise following open coding, axial coding, and selective coding, as suggested by Lindlof and Taylor [51]. The first step was open coding, in which the coder was open to all possibilities of categorizing content into different codes related to CVDs and CVRFs. After open coding, axial coding was used to regroup the codes from open coding and draw the main themes from the content. Themes were identified based on recurrence, repetition, and forcefulness [52]. Recurrence is when salient meaning appears multiple times. Repetition refers to the repeated key words, phrases, and sentences. Forcefulness is the emphasis on certain words, phrases, and sentences through underlining, increased size of print, or different colors. Finally, selective coding was used to choose representative quotes from the articles to demonstrate each theme. In reporting the results, we described the main themes and then shared some selective coding examples under each theme.

## 4. Results

**Figure 1:**
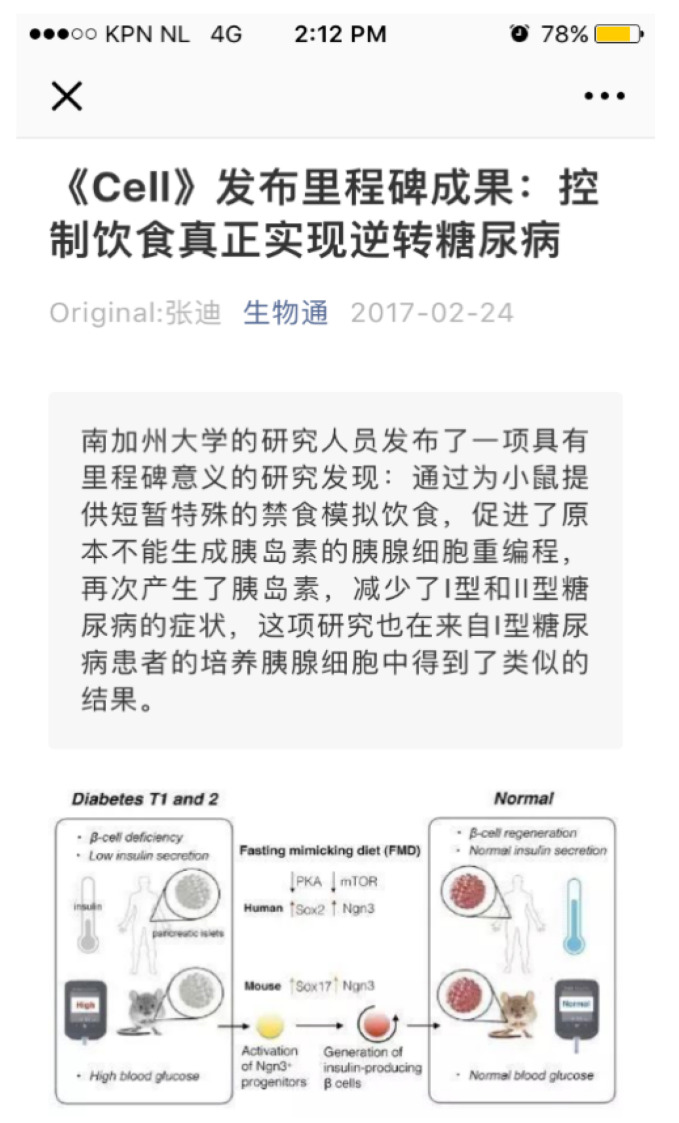
The first page of a WeChat article in our corpus.

The WeChat articles in our corpus mainly consisted of written text and pictures. The latter helped to illustrate the former and were also used to make the articles more interesting. Figure 1 shows how the first page of a typical WeChat article appears on a smartphone. It started with the Chinese text of news on a milestone research recently published in an international academic journal *Cell.* The research findings based on experiments on mice had implications on ways of using diet to control diabetes. Following the text, a figure was used to further explain and illustrate the complicated scientific findings.

Our thematic analysis of the articles revealed that they mainly included information about three related themes: how to live a healthy lifestyle, explanations of CVDs and CVRFs, and the experiences of famous people concerning these conditions. Traditional Chinese medicine in relation to CVDs also emerged as a culturally specific theme. Finally, although there was plenty of information on CVDs, none of this was targeted at the Hui people.

### 4.1. Healthy Lifestyles

Approximately 60 percent of the WeChat articles in our corpus emphasized the importance of a healthy lifestyle and provided advice on how to achieve this. The information could be clustered into two categories: a healthy diet and physical activity, with the authors of the articles assuming that many readers did not eat the right food or do any exercise.

Regardless of the different eating habits of the Han majority and Hui minority, Chinese diets in general can be regarded as both healthy, due to the amount of vegetables consumed, and unhealthy, for example when dishes are cooked with a lot of oil and salt [53]. The WeChat articles not only stressed that eating healthily can reduce CVRFs and prevent CVDs, but also contained information on appropriate diets targeted at people with different medical conditions. For example, some presented information targeting those who are currently healthy on how to reduce CVRFs or prevent CVDs through their diets, whereas others focused on the kinds of food that CVD patients should eat or avoid. An article titled “What are the diagnostic standards of hypertension, hyperlipidemia, hyperglycemia, and high uric acid? What food should be strictly avoided by patients suffering from those conditions? A comprehensive list of dos and don’ts for everyone” focused on patients suffering from different diseases and presented information about what they should not eat; it therefore included a list targeted at people with a variety of conditions. For example:

Patients with hypertension should avoid four kinds of food: 1. MSG, 2. cold drinks, 3. strong tea, 4. alcohol. Diabetes patients should eat less of these four kinds of food: 1. dragon fruit, 2. wild vegetables, 3. alcohol, 4. peanuts, melon seeds, and other nuts.

Specifically, a few articles contained detailed information on how to have a healthy lifestyle by providing recipes for making healthy food at home. These articles explained how to prepare the food, step by step, in an easy-to-understand way, making it manageable to effect the behavioral changes required for a healthy diet. In the article titled “The five most classic recipes for making eggplant more delicious than in a restaurant”, the author stated, “The vitamin E in eggplants is anti-aging and can prevent cholesterol levels in the blood from rising”. After explaining the nutritional benefits of eggplants, five recipes were demonstrated using pictures.

In general, Chinese people do not have enough exercise, leading to a rising burden of chronic diseases in the country [54]. The WeChat articles also highlighted the importance of physical activity, as this can prevent CVDs or reduce CVRFs. For example, an article titled “[Diabetes] Is diabetes ‘infectious’? How can diabetes be prevented in couples?” explained how to prevent the condition by doing sports, stating that, “Experts suggest that diabetes patients should do at least 150 min of physical activity each week… The experts emphasize that the time doing sports must be more than 20 min, so that the sugar can be consumed”.

Overall, the WeChat articles seemed to assume many Chinese people do not have a healthy diet or enough exercise. The majority of the articles aimed to promote positive behavioral changes for a healthier lifestyle.

### 4.2. The Explanations of CVDs and CVRFs

Around 80% of the WeChat articles explained CVDs and/or CVRFs, and some presumed that many readers do not know much about them. The articles highlighted some of the important characteristics or symptoms of such diseases that might be ignored; the rationale behind it was that optimal time for treatment could be missed since people were likely to ignore symptoms if they could not recognize them. A typical article titled “Five symptoms of hypertension, do not wait to treat them until you discover them” mentioned that some people ignored important symptoms and mistakenly stated, “If you don’t feel dizzy, your blood pressure isn’t high…; even though your blood pressure is high, you don’t need to deal with it if you don’t have a headache, red face, or dizziness”. This article attempted to remind people not to ignore early symptoms: “This idea is very dangerous, because most of the time when you already feel sick, it’s too late for treatment”. It also outlined the relationships between different symptoms and hypertension.

The causes, high-risk populations, and dangerous consequences of CVDs were also covered in the WeChat articles to warn those who grew up in families with a vulnerable genetic history or who had bad eating habits. The articles also contained information about effective ways to prevent or treat CVDs, to ensure that readers would not be misled by inaccurate or incorrect advice. For example, the article titled “Watch out! The six most common high blood pressure scams, many people are fooled”. highlighted six scams concerning hypertension and provided appropriate advice. The article rebutted the fake claim that “several rounds of treatment can cure high blood pressure” and explained that “the control of hypertension is a long process… treatments are adjusted based on individual cases”.

Various types of available treatment for CVDs were also discussed in the WeChat articles, with the focus mainly on medical treatment. As some patients suffer from the same disease but have very different symptoms, the articles disseminated medical information on an individual level to shed light on the appropriate treatment. A typical example was contained in an article titled “Some diabetes patients actually don’t need medicines or injections. Can I do this as well?” It discussed how patients with different types of diabetes should choose the most suitable treatment; for example, the article stated that most diabetes patients require medication: “For Type 1 diabetes patients: this is caused by insufficient insulin secretion, and so these patients must use insulin as their treatment”.

The articles not only discussed medications, but also included advertisements for such forms of treatment. Generally, these articles began with information related to using homeopathic medicines or pharmaceuticals, then relevant advertisements with web links or the QR codes of the sellers were displayed. An article titled “Greasy head and face, edema and obesity, heavy moisture? You can still eat like this!” is a typical example. It first discussed how to treat excess moisture in the body with a treatment recipe, but then suggested buying a product with the same function called “red peony powder” from a store at Tmall.com if the treatment recipe was not accessible. The inclusion of advertisements in these articles may be convenient for those who need to buy medication, but also make the articles appear to be a way of selling products instead of delivering unbiased health advice. Indeed, our analysis revealed that WeChat official accounts are not only a platform for disseminating not-for-profit CVD-related health information, but also a way for vendors to promote their medical products for financial gain.

### 4.3. Famous People’s Experiences Related to CVDs

Using famous people in persuasive messages is a common and effective persuasion strategy in health communication [55], and 17 WeChat articles in our corpus adopted this approach. In the articles we examined, these people included famous doctors or other health professionals, movie stars, singers, and politicians. The articles started by introducing the famous doctors or health professionals, and then reported their opinions or advice. A typical example could be found in the article “[Health] Everyone can live to 100, the key is to enjoy the ‘first spring’ in life! Hong Zhaoguang gives you 5 key suggestions”. This article started by introducing a famous expert on CVDs, Prof. Hong from Beijing Anzhen Hospital, who described five healthy lifestyles to help people avoid diseases and live to 100 years old. One example was: “Sentence 1: Don’t get a fat belly. The bigger your belly is, the shorter your lifespan will be!”.

**Figure 2:**
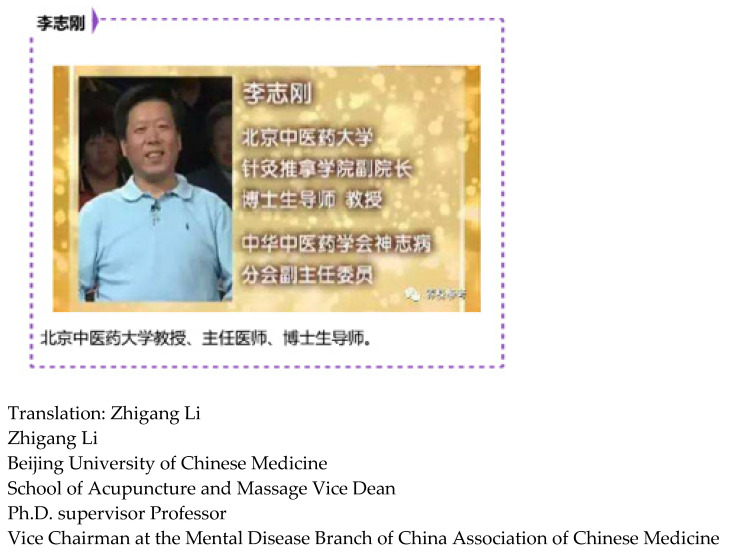
Expert introduction part in the article entitled “Stick to this action for a minute a day, and you won’t have high blood pressure, diabetes, or dementia”.

Citing a famous doctor made this article seem more reliable and more persuasive, as its readers were more likely to believe and follow suggestions made by a renowned health professional who was an expert in his field. In addition, to emphasize the authority and credibility of the famous doctors involved in the articles, some contained a professional photo next to details of his/her name, title, and current job. The article “Stick to this action for a minute a day, and you won’t have high blood pressure, diabetes, or dementia” introduced the expert with a professional photo (see Figure 2).

The WeChat articles also incorporated the experiences of famous actors, singers, and politicians. The article “Know yourself well before losing weight, so you can get twice the results with half the effort…” introduced Andy Lau, a very famous actor and singer from Hong Kong, China. The article shared how Andy Lau kept well and fit by eating healthily and exercising regularly, in order to promote information on healthy diet.

On the one hand, these WeChat official accounts used famous doctors or health professionals as credible sources to promote health information. On the other hand, the experiences of movie stars, singers, and politicians were employed as persuasive examples of how readers should live their lives.

### 4.4. Traditional Chinese Medicine in Relation to CVDs

Traditional Chinese medicine is given a great deal of attention in WeChat official accounts. Approximately one fifth of the articles in our sample contained information about this. These articles analyzed the mechanisms of diseases from a traditional Chinese medicine perspective. An example was contained in the article “Heart disease, cancer, and hypertension are all related to it! Professor in traditional Chinese medicine: A home-cooked dish to treat all”. This article stated, “The symptom of blood stasis is the cause of many diseases. Heart disease, cancer, and hypertension are all related to it”. The article then showed how to identify whether a patient had the symptom of blood stasis. In general, traditional Chinese medicine was portrayed in our sample as different from Western medicine in analyzing what causes diseases.

The WeChat articles also promoted health information related to the prevention and treatment of CVDs according to the traditional Chinese medicine perspective. This information could be clustered into two categories: how to make specific tea or soup with traditional Chinese ingredients, and acupressure massage. The aim of the recipes was to show how to prevent or treat CVDs by changing people’s health behaviors. An example was contained in an article titled “[Diabetes] What kinds of individuals are more likely to suffer from diabetes? A famous doctor couple will share secret treatment experiences!” In this article, a couple who were both traditional Chinese medicine doctors shared a prescription to control diabetes, which included special Chinese medicine ingredients such as raw jaundice, wolfberry, pueraria, gynostemma, and salvia. The article also stated, “Please pay attention as everyone’s situation is different, so you had better consult doctors and choose the proper ones”. Accordingly, although readers could make the tea or soup based on the shared recipes, they still needed to consult a doctor if they were unsure about their health circumstances. The tea and the soup, as advocated, might help prevent or relieve the symptoms of diseases, but should not replace medical treatment.

The articles also introduced information on how to prevent or treat CVDs using acupressure massage, which was usually presented in a step-by-step, easy-to-understand way. An article titled “Spend 13 seconds pressing here every day, to prevent heart disease, cervical spondylosis, stomach problems So practical!” stated, “There are some important accupoints on… people’s fingers” and described how to press them to reduce the chances of having, for example, a heart attack. The articles claimed that the function of acupressure was mainly the relief of symptoms or prevention.

## 5. Conclusions

This study only analyzed CVD-related health information in the selected articles, with the focus on whether the information was specific to the Hui. The scientific–medical validity of the content of the articles was not studied in this project. This exploratory research aimed to answer two questions. The first examined the kinds of CVD-related health information presented in WeChat official accounts, whereas the second explored whether any of this information was targeted at the Hui minority group in China.

In response to the first research question, the results revealed that the WeChat official accounts promoted information related to healthy lifestyles, especially with respect to diets and exercise. The articles claimed that having a healthy lifestyle could help people reduce CVRFs and prevent CVDs. They also presented explanations of CVDs and CVRFs. In terms of treatment, the articles’ focus was on the use of medicines, and they often included advertisements of the medicines. Famous people’s experiences with CVDs were used as a strategy to promote health, and traditional Chinese medicine likewise played an important role.

In terms of the second research question concerning whether CVD-related health content was targeted at the Hui minority, the results revealed that this was not the case.

## 6. Discussion

This study is among the first to contribute to the literature on the kinds of health information on CVDs depicted on WeChat, and if any of this is relevant to the Hui minority group. The articles on the WeChat official accounts we examined emphasized a healthy lifestyle in the form of a good diet and physical activity, probably because many Chinese people do not eat healthily or do enough exercise; for example, meat plays a special role in Chinese diets and it is one of the most important parts of a daily meal [56]. The articles contained a lot of information on CVDs and CVRFs. Many of the articles stated that people misunderstood these conditions, reflecting earlier findings that many Chinese adults did not properly understand health information [57]. When communicating advice related to the use of medicines, some of the pieces also included advertisements for medication. This might be convenient for those who needed it, but also turned the articles into commercials instead of just service articles for conveying health information. The inclusion of advertisements is related to the purposes of the official accounts: Some aim to benefit financially by promoting products, whereas others have a not-for-profit ethos and a goal of communicating important health information to the public. The complicated healthcare system in China means that some people earn money from prescribing medicines. This also happened in our study corpus: Some WeChat official accounts promoted medicines in the articles and aimed to make a profit. As a consequence, WeChat users getting health information from official accounts must judge their goals critically.

The results of this study are undoubtedly partially related to the two kinds of medical systems in China: traditional Chinese and Western. Although some Chinese people do not use the former, our findings show that it is not marginalized because of the existence of the latter. Traditional Chinese medicine played an important role in the articles on the WeChat official accounts. Most of the information related to this system focused on how to prevent and treat CVDs or reduce CVRFs using its interventions. The presence of both Western and traditional Chinese perspectives in the articles is related to Keji and Hao’s [58] findings that each system may have its advantages for the Chinese population in terms of the prevention and treatment of diseases and therefore may provide benefits to the other.

The WeChat official accounts also shared famous people’s experiences to communicate health information. Previous research shows that doctors or health professionals are regarded as trustworthy and credible sources [59,60]. Consequently, the information they provide can be persuasive. The prominence of stories by movie stars, singers, and politicians suggests that it is also assumed that such people can influence health beliefs and practices [55].

The results of our analysis of these WeChat articles confirm the value of qualitative content analysis, as this enabled us to determine the kinds of health information on CVDs conveyed by the official accounts. We acknowledge that the research has some limitations, in particular the size of the corpus we selected. Nevertheless, having used the selection criterion of an access frequency of more than 50,000 times (5W+) makes us confident that the information shared in these official accounts is circulated widely by WeChat users. Future research could take a quantitative approach to analyze a large sample of WeChat articles, which could provide more information on whether and how less circulated WeChat articles provide CVD-related health information targeting the Hui specifically. It is also meaningful to examine health information in articles in Hui ethnic official WeChat accounts.

Overall, the patterns in our results indicate that official accounts are an effective way of communicating specific health information [8]. The advice related to CVDs and CVRFs is rich and diverse and warrants future studies about the specifics of health content on WeChat official accounts. The lack of information specifically targeted at the Hui people does not mean that this information is not important for them; instead, the opposite is true, as previous research has indicated that a healthy lifestyle, as promoted in our corpus of articles, should be available to this minority group [9]. Against this backdrop, WeChat official accounts could be an effective platform for health promotors to disseminate CVD-related health information to help the Hui adopt a healthier lifestyle.

## Data Availability

The data of this research is concerned with 108 articles that were published on WeChat official accounts from December 2016 to February 2017. In February 2018, the authors entered the terms “heart attack”, “CVDs”, “diabetes”, “obesity”, “hypertension”, and “hyperlipidemia” in Chinese in the search engine of the Qingbo Big Data website (http://www.gsdata.cn/, accessed on 9 May 2018). These 108 articles were public online when we conducted this study. These 108 articles were downloaded in the PDF format and stored by the corresponding author.

## Notes

WeChat articles cited in the paper are listed in the Appendix A. All quotes were translated as literally as possible from Mandarin to English.

## Appendix A

**Table A1:** Overview of the WeChat Articles Shown in the Results.

#	Name of the Article (English)	Name of the Article (Chinese)	Published Date	Published Account (English)	Published Account (Chinese)
**1**	What are the diagnostic standards of hypertension, hyperlipidemia, hyperglycemia, and high uric acid? What food should be strictly avoided by patients suffering from those conditions? A comprehensive list of dos and don’ts for everyone.	高血压、高血脂、高血糖、高尿酸的标准是啥，最不能吃啥？这个超全的清单早该人手一份了！)	3 February 2017	Sir Run Run Shaw Hospital	邵逸夫医院
**2**	The five most classic recipes for making eggplant more delicious than in a restaurant.	茄子最经典的5种做法，比饭店还好吃！	2 January 2017	Tian tian tian tian’s life note	甜甜 甜甜的生活手札
**3**	[Diabetes] Is diabetes ‘infectious’? How can diabetes be prevented in couples?	【糖尿病】糖尿病会“传染”？如何防止夫妻糖尿病的发生	23 February 2017	Xiao bian yuan bao BTV yang sheng tang	小编元宝 BTV 养生堂
**4**	Five symptoms of hypertension, do not wait to treat them until you discover them.	高血压的5大症状，别等发现了才去治	17 January 2017	Ding dang ding xiang Doctor	丁当 丁香医生
**5**	Watch out! The six most common high blood pressure scams, many people are fooled.	千万注意！这 6 个最常见的高血压骗局，很多人上当	3 January 2017	Wang xin health headlines	王锌 健康头条
**6**	Some diabetes patients actually don’t need medicines or injections. Can I do this as well?	有的糖尿病人，居然可以不吃药不打针，我可以这样吗？	10 January 2017	Xu nai jia health headlines	徐乃佳 健康头条
**7**	Greasy head and face, edema and obesity, heavy moisture? You can still eat like this!	头脸油腻、水肿肥胖、湿气重？你还可以这样吃！	8 January 2017	Coming from star	来自星星
**8**	[Health] Everyone can live to 100, the key is to enjoy the ‘first spring’ in life! Hong Zhaoguang gives you 5 key suggestions.	【健康】人人可活100岁，关键是过好人生 ‘第一春’！洪昭光送你5句话	27 February 2017	People’s Daily	人民日报
**9**	Stick to this action for a minute a day, and you won’t have high blood pressure, diabetes, or dementia.	每天坚持这个动作1分钟，高血压，糖尿病，老年痴呆都与你无缘〜	25 February 2017	New old people	新老人
**10**	Know yourself well before losing weight, so you can get twice the results with half the effort…	减肥之前要有一个正确的自我认知，这样才能事半功倍…	22 February 2017	James’ kitchen	詹姆士的厨房
**11**	Heart disease, cancer, and hypertension are all related to it! Professor in traditional Chinese medicine: A home-cooked dish to treat all.	心病，癌症，高血压都与它有关！中医教授: 一道家常菜，通通搞定	15 January 2017	New old people	新老人
**12**	[Diabetes] What kinds of individuals are more likely to suffer from diabetes? A famous doctor couple shares secret treatment experiences!	【糖尿病】什么样的人易得糖尿病？名医夫妻揭秘治病经验方！	4 February 2017	Xiao bian yuan bao BTV yang sheng tang	小编元宝 BTV养生堂
**13**	Spend 13 s pressing here every day to prevent heart disease, cervical spondylosis, stomach problems… So practical!	每天花13秒按这，远离心脏病、颈椎病、胃病……太实用了！	10 January 2017	New old people	新老人
